# Bayesian Tensor Decomposition for Clustering Latent Symptom Profiles for Verbal Autopsy Data

**DOI:** 10.1002/sim.70475

**Published:** 2026-03-03

**Authors:** Yu Zhu, Zehang Richard Li

**Affiliations:** ^1^ Department of Statistics University of California, Santa Cruz California USA

**Keywords:** Bayesian hierarchical model, cause‐of‐death classification, mortality quantification, probabilistic tensor decomposition, verbal autopsy

## Abstract

Cause‐of‐death data is fundamental for understanding population health trends and inequalities as well as designing and evaluating public health interventions. A significant proportion of global deaths, particularly in low‐ and middle‐income countries (LMICs), do not have medically certified causes assigned. In such settings, verbal autopsy (VA) is a widely adopted approach to estimate disease burdens by interviewing caregivers of the deceased. Recently, latent class models have been developed to model the joint distribution of symptoms and perform probabilistic cause‐of‐death assignment. A large number of latent classes are usually needed in order to characterize the complex dependence among symptoms, making the estimated symptom profiles challenging to summarize and interpret. In this paper, we propose a flexible Bayesian tensor decomposition framework that balances the predictive accuracy of the cause‐of‐death assignment task and the interpretability of the latent structures. The key to our approach is to partition symptoms into groups and model the joint distributions of group‐level symptom sub‐profiles. The proposed methods achieve better predictive accuracy than existing VA methods and provide a more parsimonious representation of the symptom distributions. We show our methods provide new insights into the clustering patterns of both symptoms and causes using the PHMRC gold‐standard VA dataset.

## 1 Introduction

Understanding cause‐of‐death distributions is vital for assessing the health status and needs of a population, particularly in low‐resource settings where data scarcity often hampers public health efforts. In many low‐ and middle‐income countries (LMICs), a substantial proportion of deaths occur outside of medical facilities, leaving these deaths unregistered and without medically certified causes of death. To bridge this information gap, verbal autopsy (VA) has emerged as a widely adopted tool, providing an alternative means of cause‐of‐death determination through structured interviews with a deceased individual's family or caregiver. VA helps infer causes of death by collecting data on demographics, symptoms, and circumstances preceding death, especially in settings lacking complete civil registration and vital statistics systems [1, 2, 3].

The process of assigning causes of death using VAs traditionally relies on physicians reviewing the collected data and assigning likely causes of death manually. While effective, physician‐based VA assessment is resource‐intensive, challenging to scale, and often impractical for routine surveillance. As a result, algorithmic and statistical methods have gained traction as cost‐effective, scalable alternatives for automated cause‐of‐death determination. Early VA cause‐of‐death assignment methods usually assume that symptoms are conditionally independent given the underlying cause of death. This simplifying assumption reduces model complexity and computation time, making these algorithms more accessible for large‐scale use in low‐resource settings [4, 5, 6]. However, real‐world symptom patterns usually exhibit complex dependence relationships that are crucial for understanding disease risks and accurate cause‐of‐death assignment. Recent work on VA analysis largely focuses on relaxing this assumption. Kunihama et al. [7] and Moran et al. [8] proposed latent Gaussian factor models to account for symptom dependence and their association with demographic covariates. Li et al. [9] proposed a latent Gaussian graphical model to characterize latent conditional independence relationships across symptoms. One of the main limitations of the latent Gaussian approach to modeling symptom dependence is that the inferred dependence structures cannot be easily interpreted on the scale of the observed discrete response variables. The latent Gaussian models are also computationally more expensive to estimate. Li et al. [10] and Wu et al. [11] addressed these challenges by developing a latent class model framework for VA, where the conditional distribution of binary symptoms given each cause of death is approximated with a finite mixture model. The symptoms are assumed to be conditionally independent given the individual‐level latent class membership. The parameters of the latent representation can then be interpreted as latent symptom profiles given each cause of death, a concept known as the symptom‐cause‐information (SCI) in the VA literature [12].

There are two main challenges with latent class analysis introduced in Li et al. [10] and Wu et al. [11]. First, the classical latent class model framework relies on a single latent variable to cluster observations. Deaths assigned to the same cluster are assumed to have independent and identical symptom distributions. When the assumed number of latent classes is small, the flexibility of the model formulation is limited and the model may not capture nuanced symptom dependence structures for more complex causes of death. On the other hand, models with a large number of latent classes increase the risk of overfitting to the noisy VA data and can degrade the classification performance for the cause‐of‐death assignment. Second, without structural assumptions, standard latent class analysis usually leads to similar latent class profiles on VA data. This phenomenon is known as weak separation [13] and is common in high‐dimensional latent class analysis. The LCVA model proposed in Li et al. [10] mitigates this issue by assuming the latent symptom profiles share similar elements on most dimensions and differ for only a sparse set of symptoms. However, as we illustrate in Section 2, when the similarity across latent profiles are induced because of symptom clustering, the sparsity assumption can be ineffective. The weak separation of latent classes makes the characterization and interpretation of high‐dimensional symptom profiles difficult.

In this paper, we propose a unified framework using hierarchical tensor decomposition that addresses both limitations. In addition to clustering deaths, we also perform simultaneous symptom clustering. Specifically, we propose two new methods for modeling VA data using group‐wise PARAFAC decomposition and collapsed Tucker (c‐Tucker) decomposition [14]. Both models partition the high‐dimensional symptoms into a small number of groups and construct the full symptom profiles by combining group‐specific symptom sub‐profiles. Compared to standard latent class models, our models allow us to capture a wider range of dependence structures among symptoms and causes while using a smaller number of parameters. The symptom groups also provide a more interpretable and parsimonious summary of the symptom‐cause relationship. We develop a scalable estimation strategy using Markov chain Monte Carlo (MCMC). Finally, we demonstrate the effectiveness of our model on VA data, showing that the tensor decomposition approach improves both the accuracy of cause‐of‐death assignments and the interpretability of the latent representations.

The rest of the paper is organized as follows. Section 2 reviews tensor decomposition models in the VA literature. Section 3 proposes two new tensor decomposition approaches to model symptom distribution. Section 4 describes posterior inference using MCMC. Section 5 evaluates the proposed approach with synthetic data and demonstrates the improved performance in cause‐of‐death assignment from the proposed models compared to existing methods. Section 6 describes an in‐depth analysis of a real‐world VA dataset using the proposed models. Section 7 concludes with a discussion and future work.

## 2 Tensor Decomposition for VA Data

Let $X_{i}\in {\left\{0,1\right\}}^{p}$ denote the p‐dimensional vector of binary signs/symptoms collected by VA and $Y_{i}\in \{1,\ldots ,C\}$ denote the reference cause of death for the ith death. In this paper, we consider the scenario where we have access to a training dataset where both X and Y are observed and inference is needed for a target dataset where we only observe X. Let $D_{i}=1$ indicates the ith death is in the training dataset and $D_{i}=0$ the target dataset. The goals of cause‐of‐death assignment using VA data usually include two components: Individual cause‐of‐death classification for deaths without a reference cause, that is, predicting p(Y|X), and the estimation of cause‐specific mortality fractions (CSMF), that is, p(Y), in the target dataset. We factorize the joint distribution p(X,Y)=p(Y)p(X|Y) following the convention VA modeling [10, 15]. This factorization corresponds more naturally to the data‐generating process, where the majority of signs and symptoms can be treated as consequences of the underlying disease. It also allows direct estimation of different p(Y) across training and target datasets.

The key to probabilistic VA models is the modeling of the high‐dimensional conditional probability tensor p(X|Y). Most existing VA models can be viewed as model‐based decompositions of these high‐dimensional probability tensors. Tensor decompositions have been successfully applied in various fields, including medical imaging, genomics, social sciences, and recommendation systems. Two most widely used models in the literature are PARAFAC decomposition [16, 17] and the Tucker decomposition [18]. The Tucker decomposition has not been explored in the VA literature due to its computational complexity, thus we leave our review in the Supporting Information. The PARAFAC decomposition, on the other hand, has been commonly used in VA modeling, and is directly relevant to our proposal in this paper. The PARAFAC decomposition decomposes a tensor into a sum of rank‐one tensors, capturing the interactions between multiple modes of the data. The K‐component element‐wise non‐negative PARAFAC decomposition of the probability tensor $p(X_{i1}=x_{1},\ldots ,X_{ip}=x_{p}|Y_{i}=c)$ assumes

(1) $$\begin{array}{ll} p(X_{i1}=x_{1},\ldots ,X_{ip}=x_{p}|Y_{i}=c) & =\overset{K}{\underset{k=1}{\sum }}\lambda_{ck}\overset{p}{\underset{j=1}{\prod }}\phi_{ckj}^{x_{j}}{\left(1-\phi_{ckj}\right)}^{1-x_{j}}. \end{array}$$

Both λ and ϕ are non‐negative, and $\sum_{k=1}^{K}\lambda_{ck}=1$ for c=1,…,C. The latent class model in Wu et al. [11] is a hierarchical version of the probabilistic PARAFAC decomposition with multiple training datasets. The model in Li et al. [10] uses a sparse PARAFAC decomposition [19] by introducing spike‐and‐slab priors to ϕ. Furthermore, when K=1, the decomposition reduces to assuming symptoms are conditionally independent given each cause of death, and it results in the widely used InSilicoVA algorithm [5]. As $\phi_{c}$ characterizes K sets of independent Bernoulli distributions from which the symptoms are generated given the cth cause of death, we refer to $\phi_{c}$ as the symptom profiles for the cth cause.

## 3 Dimension‐Grouped Tensor Decomposition of VA Symptoms

A salient feature of VA data is that many symptoms/signs are naturally organized in groups. For instance, for breathing‐related issues, VA questionnaires typically ask about a series of related questions, such as whether the person had fast breathing, prolonged trouble breathing, intermittent trouble breathing, increased trouble breathing in certain positions, and so forth. VA questionnaires also usually include demographic indicators and indicators associated with risk factors such as drinking and smoking. In order to capture this grouping feature among symptoms, we consider two tensor decomposition models: Grouped independent PARAFACs and c‐Tucker decomposition. Both formulations were studied in Johndrow et al. [14].

Assuming the symptoms can be divided into r groups, where 1<r<p, one straightforward extension to the PARAFAC decomposition is to model each group independently with multiple PARAFAC decompositions. We refer to this model as r‐group independent PARAFACs. Let $s_{j}\in \{1,\ldots ,r\}$ denote the group membership indicator for the jth symptom. The r‐group independent PARAFACs model assumes

(2) $$\begin{array}{ll} p(X_{i1} & =x_{1},\ldots ,X_{ip}=x_{p}|Y_{i}=c)\\ & =\overset{r}{\underset{s=1}{\prod }}p(\{X_{ij}=x_{j}:s_{j}=s\}|Y_{i}=c), \end{array}$$

(3) $$\begin{array}{ll} p(\{X_{ij} & =x_{j}:s_{j}=s\}|Y_{i}=c)\\ & =\overset{K_{s}}{\underset{k_{s}=1}{\sum }}\lambda_{ck_{s}}\underset{j:s_{j}=s}{\prod }\phi_{ck_{s_{j}},j}^{x_{j}}{\left(1-\phi_{ck_{s_{j}},j}\right)}^{1-x_{j}}, \end{array}$$

where $K_{s}$ is the number of latent classes in the sth symptom group. In this work, as our main goal is using the decomposition to approximate the joint distribution of symptoms, rather than identifying optimal latent clusters, we treat $K_{1}=\cdots =K_{r}$ and drop the subscript. Similar to the PARAFAC decomposition, the dependence of symptoms within the same group can be arbitrarily flexible with a large enough K. The symptoms from different groups are assumed to be independent a prior.

The between‐group conditional independence assumption is likely too rigid in VA modeling, since groups of symptoms and risk factors are likely dependent given the underlying disease. To further introduce dependence among the groups, we adopt the c‐Tucker formulation in Johndrow et al. [14] and model the mixing weights λ using another non‐negative PARAFAC decomposition. That is,

(4) $$\begin{array}{ll} p(X_{i1} & =x_{1},\ldots ,X_{ip}=x_{p}|Y_{i}=c)\\ & =\overset{K_{1}}{\underset{k_{1}=1}{\sum }}\ldots \overset{K_{r}}{\underset{k_{r}=1}{\sum }}\lambda_{ck_{1},\ldots ,ck_{r}}\overset{p}{\underset{j=1}{\prod }}\phi_{ck_{s_{j}},j}^{x_{j}}{\left(1-\phi_{ck_{s_{j}},j}\right)}^{1-x_{j}}, \end{array}$$

(5) $$\begin{array}{ll} \lambda_{ck_{1},\ldots ,ck_{r}} & =\overset{h}{\underset{l=1}{\sum }}\nu_{cl}\overset{r}{\underset{s=1}{\prod }}\psi_{clk_{s}}. \end{array}$$

The c‐Tucker decomposition reduces to the r‐group independent PARAFACs when h=1 and the standard PARAFAC when h=r=1. On the other hand, when r=p, it is equivalent to the standard Tucker decomposition. Therefore, it provides an appealing way to control the complexity of the latent structure. Similar to the r‐group PARAFAC model, we make the simplifying assumption that $K_{1}=...=K_{r}$ and drop the subscript.

**FIGURE 1:**
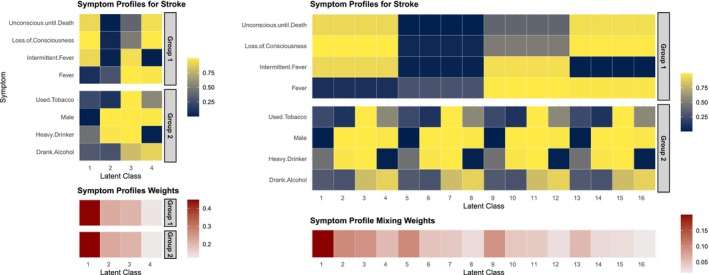
Posterior mean of latent parameters for stroke estimated from the c‐Tucker model in one synthetic dataset. Left two panels are the selected symptoms profile $\phi_{c}$ by latent class from 2 symptom groups (top), ordered by the latent class weights $p(Z_{is}=k|Y_{i}=c)=\sum_{l=1}^{h}\nu_{cl}\psi_{clsk}$ (bottom). Right two panels are the expanded version of selected symptoms profile ϕ (top) if the profiles are parameterized under standard PARAFAC decomposition, and the equivalent mixing weights, that is, $p(Z_{is_{1}}=k_{1},Z_{is_{2}}=k_{2}|Y_{i}=c)$ for $s_{1},s_{2}\in \{1,2\}$ and $k_{1},k_{2}\in \{1,\ldots ,4\}$.

In both dimension‐grouped decompositions, the symptom profile $\phi_{c}$ is still a K by p matrix, but a different latent class is assigned for each symptom group. To illustrate the expressiveness of the symptom profiles estimated by the two dimension‐grouped factorization models, Figure 1 shows an example of a subset of latent symptom profiles ϕ and the corresponding weights λ for deaths due to stroke using the c‐Tucker model and the data described in Section 6. The combinations of these two group‐level sub‐profiles $\left\{\phi_{c,k,j}:k\in 1,\ldots ,K,s_{j}=1\right\}$ and $\left\{\phi_{c,k,j}:k\in 1,\ldots ,K,s_{j}=2\right\}$ can be equivalently represented by the standard PARAFAC model with $K^{2}$ separate latent profiles. The right panel of Figure 1 illustrates this expanded representation. With r symptom groups and K latent classes, the symptom profiles under the dimension‐grouped factorization are parameterized by pK parameters, whereas for the standard PARAFAC, it requires $pK^{r}$ parameters to characterize the same dependence structure, with many repeated elements. In practice, with a finite number of observations, the PARAFAC model will likely not fully utilize $K^{r}$ latent classes but will approximate it with a smaller rank instead due to prior shrinkage. This leads to a loss of information on the symptom profiles and can make the resulting latent representation more difficult to interpret.

### 3.1 Bayesian Hierarchical Representation

To connect the r‐group independent PARAFACs and c‐Tucker decomposition to the latent class models used for cause‐of‐death assignment, we introduce latent indicators and equivalently represent the conditional probability tensor decomposition using Bayesian hierarchical latent variable models. Under both models, we introduce r latent indicators for each death, where $Z_{is}\in \{1,\ldots ,K\}$ specifies latent class membership in the sth symptom group for the ith death. Let $s_{cj}\in \{1,\ldots ,r\}$ denote the group membership for the jth symptom among death due to cth cause. Then the r‐group independent PARAFACs decomposition can be expressed by

(6) $$\begin{array}{ll} Z_{is}|Y_{i}=c & ∼\text{Cat}(\lambda_{cs}), \end{array}$$

(7) $$\begin{array}{ll} X_{ij}|Y_{i}=c,Z_{is_{cj}}=k & ∼\text{Bern}(\phi_{ckj}). \end{array}$$

For the c‐Tucker model, we introduce another latent categorical variable $H_{i}\in \{1,..,h\}$ to facilitate the dependence among the group‐level indicators $Z_{is}$. That is,

(8) $$\begin{array}{ll} H_{i}|Y_{i}=c & ∼\text{Cat}(\nu_{c}), \end{array}$$

(9) $$\begin{array}{ll} Z_{is}|H_{i}=l,Y_{i}=c & ∼\text{Cat}(\psi_{cls}), \end{array}$$

(10) $$\begin{array}{ll} X_{ij}|Y_{i}=c,Z_{is_{cj}}=k & ∼\text{Bern}(\phi_{ckj}). \end{array}$$

Finally, we complete the model specification with the priors for Y and the latent parameters. We assume the CSMFs in the training and target datasets can differ, but the conditional distribution of symptoms given causes remains the same, that is, there is label shift (also known as prior shift) between the joint distributions p(X,Y) in the two datasets [20]. This is usually a reasonable assumption when both datasets are collected on the same population. Specifically, for g∈{0,1}, we let

(11) $$\begin{array}{ll} Y_{i}|D_{i}=g & ∼\text{Cat}(\pi^{\left(g\right)}) \end{array}$$

(12) $$\begin{array}{ll} \pi^{\left(g\right)} & ∼\text{Dir}(\alpha_{1},\ldots ,\alpha_{C}). \end{array}$$

Since we are interested in extracting a small number of groups among symptoms, we use a Dirichlet prior $\lambda_{cs}∼\text{Dir}(a_{s1},\ldots ,a_{sK})$ under the r‐group independent PARAFACs. For the c‐Tucker model, we let $\psi_{cls}∼\text{Dir}(b_{s1},\ldots ,b_{sK})$, $\nu_{c}∼\text{Dir}(1/h,\ldots ,1/h)$. These Dirichlet priors may be replaced with stick‐breaking priors to further encourage sparsity when the latent dimensions are higher. For the response probabilities, we use non‐informative Beta priors, that is, $\phi_{ckj}∼\text{Beta}(1,1)$.

As for the symptom groups, they may be predetermined if there exists expert knowledge of symptom grouping. In this paper, we adopt a more general data‐driven approach and estimate the symptom grouping instead. We assume the following priors on the group memberships,

(13) $$\begin{array}{ll} s_{cj} & ∼\text{Cat}(\xi_{c}), \end{array}$$

(14) $$\begin{array}{ll} \xi_{c} & ∼\text{Dir}(1/r,\ldots ,1/r). \end{array}$$

## 4 Posterior Computation

Posterior distribution of the model parameters can be easily approximated using Gibbs sampling. We describe the MCMC steps for the c‐Tucker model first and then the modifications to the r‐group independent PARAFACs model.

1. Sample $Y_{i}|X_{i},s,\pi ,\phi ,\psi ,\nu$ for unknown $Y_{i}$ with
$$\begin{array}{ll} p(Y_{i}=c|X_{i},s,\pi ,\phi ,\psi ,\nu ) & \propto \pi_{c}^{\left(g\right)}\overset{K}{\underset{k_{1}=1}{\sum }}\ldots \overset{K}{\underset{k_{r}=1}{\sum }}\left(\overset{h}{\underset{l=1}{\sum }}\nu_{cl}\overset{r}{\underset{s=1}{\prod }}\psi_{clsk_{s}}\right)\\ & \;\overset{p}{\underset{j=1}{\prod }}\phi_{ck_{s_{cj}}j}^{X_{ij}}{\left(1-\phi_{ck_{s_{cj}}j}\right)}^{1-X_{ij}} \end{array}$$
2. Sample $Z_{is}|H_{i}=l,Y_{i}=c,X_{i},s,\phi ,\psi$ for i=1,…,n, s=1,…,r with
$$\begin{array}{ll} & p(Z_{is}=k|H_{i}=l,Y_{i}=c,X_{i},s,\phi ,\psi )\\ & \;\propto \psi_{clsk}\overset{p}{\underset{j:s_{cj}=s}{\prod }}\phi_{ckj}^{X_{ij}}{\left(1-\phi_{ckj}\right)}^{1-X_{ij}} \end{array}$$
3. Sample $H_{i}|Y_{i}=c,Z_{is},\psi ,\nu$ for l=1,…,h with
$$\begin{array}{l} p(H_{i}=l|Y_{i}=c,Z_{is},\psi ,\nu )\propto \nu_{cl}\overset{r}{\underset{s=1}{\prod }}\psi_{cls,Z_{is}} \end{array}$$
4. Sample $\pi^{\left(g\right)}|Y$ with
$$\begin{array}{l} \pi^{\left(g\right)}|Y∼\text{Dir}\left(\alpha_{1}+\overset{n_{g}}{\underset{i=1}{\sum }}1_{\left\{Y_{i}=1\right\}},\ldots ,\alpha_{C}+\overset{n_{g}}{\underset{i=1}{\sum }}1_{\left\{Y_{i}=C\right\}}\right). \end{array}$$
5. Sample $\nu_{c}|Y,H$ with
$$\begin{array}{ll} & \nu_{c}|Y∼\text{Dir}\\ & \;\left(1/h+\overset{n}{\underset{i=1}{\sum }}1_{\left\{Y_{i}=c,H_{i}=1\right\}},\ldots ,1/h+\overset{n}{\underset{i=1}{\sum }}1_{\left\{Y_{i}=c,H_{i}=h\right\}}\right). \end{array}$$
6. Sample $\psi_{cls}|Y,H,Z$ with
$$\begin{array}{ll} & \psi_{cls}|Y∼\text{Dir}\\ & \;\left(b_{s1}+\overset{n}{\underset{i=1}{\sum }}1_{\left\{Y_{i}=c,H_{i}=l,Z_{is}=1\right\}},\ldots ,b_{sK}+\overset{n}{\underset{i=1}{\sum }}1_{\left\{Y_{i}=c,H_{i}=l,Z_{is}=K\right\}}\right). \end{array}$$
7. Sample $\phi_{ckj}|Y,X,Z,s$, for c=1,…,C, k=1,…,K, j=1,…,p, with
$$\begin{array}{ll} & \phi_{ckj}|Y,X,Z,s∼\text{Beta}\\ & \;\left(a_{\phi }+\overset{n}{\underset{i=1}{\sum }}1_{\left\{Y_{i}=c,Z_{is_{cj}}=k,X_{ij}=1\right\}},b_{\phi }+\overset{n}{\underset{i=1}{\sum }}1_{\left\{Y_{i}=c,Z_{is_{cj}}=k,X_{ij}=0\right\}}\right). \end{array}$$
8. Sample $s_{cj}|Y,X,Z,\phi ,\xi$ for j=1,…,p, s=1,…,r, c=1,…,C with
$$\begin{array}{ll} & p(s_{cj}=s|Y,X,Z,\phi ,\xi )\\ & \;=\frac{\xi_{cs}\prod_{i:Y_{i}=c}^{n}\phi_{c,Z_{is},j}^{X_{ij}}{\left(1-\phi_{c,Z_{is},j}\right)}^{1-X_{ij}}}{\sum_{s=1}^{r}\xi_{cs}\prod_{i:Y_{i}=c}^{n}\phi_{c,Z_{is},j}^{X_{ij}}{\left(1-\phi_{c,Z_{is},j}\right)}^{1-X_{ij}}.} \end{array}$$
9. Sample $\xi_{c}|s_{c}$ for c=1,…,C with
$$\begin{array}{l} \xi_{c}|s_{c}∼\text{Dir}\left(1/r+\overset{p}{\underset{j=1}{\sum }}1_{\left\{s_{cj}=1\right\}},\ldots ,1/r+\overset{p}{\underset{j=1}{\sum }}1_{\left\{s_{cj}=r\right\}}\right). \end{array}$$

As for the r‐group independent PARAFACs, the sampler proceeds with the following.

1. Sample $Y_{i}|X_{i},s,\pi ,\phi ,\psi ,\nu$ for unknown $Y_{i}$ with
$$\begin{array}{ll} & p(Y_{i}=c|X_{i},s,\pi ,\phi ,\psi ,\nu )\\ & \;\propto \pi_{c}^{\left(g\right)}\overset{r}{\underset{s=1}{\prod }}\overset{K}{\underset{k=1}{\sum }}\lambda_{csk}\overset{p}{\underset{j:s_{j}=s}{\prod }}\phi_{ckj}^{X_{ij}}{\left(1-\phi_{ckj}\right)}^{1-X_{ij}}. \end{array}$$
2. Sample $Z_{is}|Y_{i}=c,X_{i},s,\phi ,\lambda$ for i=1,…,n, s=1,…,r with
$$\begin{array}{l} p(Z_{is}=k|Y_{i}=c,X_{i},s,\phi ,\lambda )\propto \lambda_{csk}\overset{p}{\underset{j:s_{j}=s}{\prod }}\phi_{ckj}^{X_{ij}}{\left(1-\phi_{ckj}\right)}^{1-X_{ij}}. \end{array}$$
3. Sample $\lambda_{cs}|Y,Z$ with
$$\begin{array}{ll} & \lambda_{cs}|Y∼\text{Dir}\\ & \;\left(b_{s1}+\overset{n}{\underset{i=1}{\sum }}1_{\left\{Y_{i}=c,Z_{is}=1\right\}},\ldots ,b_{sK}+\overset{n}{\underset{i=1}{\sum }}1_{\left\{Y_{i}=c,Z_{is}=K\right\}}\right). \end{array}$$
4. Sample $\pi^{\left(g\right)}$, $\phi_{ckj}$, $s_{cj}$ and $\xi_{c}$ in the same way as the sampler for the c‐Tucker model.

## 5 Simulation Study

We start with a simulation study using synthetic data to evaluate the performance of different tensor decomposition models in terms of cause‐of‐death assignment. We consider C=20 causes of death, p=80 symptoms, and a total of 3000 deaths where the training data consist of 2000 labeled VAs and target data consist of 1000 unlabeled VAs. For each training‐target pair, we generate $\pi^{\left(g\right)}∼\text{Dir}(1,\ldots ,1)$ for g=0 and 1 independently. The symptoms X are generated according to the c‐Tucker model with latent dimensions K=3, r=5 and h=3. For each cause c and latent group l, we set $\nu_{cl}=1/h$, ensuring equal contribution across groups. We construct the s matrix where each row consists of r sequential groups arranged in a cyclic pattern:

$$\begin{array}{l} s_{cj}=\left\lfloor \frac{(j-(c-1))\;\text{mod}\;p}{g}\right\rfloor +1, \end{array}$$

where g=p/r, 1≤c≤C, and 1≤j≤p. We consider two scenarios of generating the latent class memberships as follows:

1. Scenario I: We generate data according to the proposed c‐Tucker model. We consider the more realistic situation where the concentrations of latent classes can vary considerably across causes and symptom groups. We let $\psi_{cls}∼\text{Dir}(\beta_{cls}1_{K})$ for each c∈{1,…,C}, l∈{1,…,h}, and s∈{1,…,r}, where the overall concentration parameters $\beta_{cls}$ are independently drawn from a discrete uniform distribution on [1,10].
2. Scenario II: We generate data according to a misspecified c‐Tucker model where additional distribution shift exists between the training and target dataset in terms of the symptom distribution conditional on causes. In this case, denote $p(Z_{is}=k|H_{i}=l,Y_{i}=c,D_{i}=g)=\psi_{clsk}^{\left(g\right)}$. We simulate $\psi_{cls}^{\left(g\right)}∼\text{Dir}(\beta_{cls}^{\left(g\right)}1_{K})$ and $\beta_{cls}^{\left(g\right)}$ are sampled from independently drawn from a discrete uniform distribution on [1,10]. In this scenario, all models are severely misspecified as p(X|Y) can be different across the training and target data, depending on the sampled mixing weights ψ.

Finally, to mimic the low signal‐to‐noise ratio in real VA datasets, we allow only a small set of the symptoms to be informative to cause‐of‐death classification. More specifically, we let

$$\begin{array}{ll} & X_{ij}|Z_{is_{cj}}=k∼\text{Bern}(\phi_{kj}),\;\phi_{kj}∼\text{Beta}(1,1),\text{if}s_{cj}\in \{1,2\},\\ & X_{ij}|Y_{i}=c,Z_{is_{cj}}=k∼\text{Bern}(\phi_{ckj}),\\ & \phi_{ckj}∼\text{Beta}(1,1),\text{if}s_{cj}\in \{3,4,5\}. \end{array}$$

In both scenarios, we generate 50 synthetic datasets and fit the c‐Tucker and the r‐group independent PARAFACs models. In the simulation study, we fix the latent dimensions to be the same as in the data generating process, that is, K=3,r=5, and h=3. We included additional simulation results with misspecified latent dimensions in the Supporting Information, and discuss practical choices of latent dimensions later in the analysis of real‐world VA data.

We compare the models with the standard PARAFAC with different numbers of latent classes, K=5,10, and 15. As discussed before, the standard PARAFAC corresponds to the c‐Tucker model with h=r=1. We also include comparisons with the LCVA algorithm described in Li et al. [10]. LCVA is based on a version of sparse PARAFAC decomposition of p(X|Y) and has been shown to outperform other existing VA methods. Varying the number of latent classes K in LCVA yields patterns similar to those in the PARAFAC model, with differences being less pronounced because the shrinkage priors in LCVA encourage more latent classes to collapse to similar values. Therefore, we only present a single LCVA specification, using K=10 following the recommendation in Li et al. [10]. Since label shift is assumed for the rest of the models in consideration, we only compare with the single‐domain variation of LCVA, where p(Y) is treated to be different but p(X|Y) is set to be the same across training and target datasets. It should be noted that LCVA implements the cause‐of‐death assignment in two stages, which is slightly different from the rest of the models in terms of the estimation procedure. For each dataset, we fit the proposed model and the PARAFAC model by running MCMC for 3000 iterations, with the first 1000 iterations discarded as burn‐in. For the LCVA, we follow Li et al. [10] and run three parallel chains of MCMC for 4000 iterations with the first 1000 iterations discarded as burn‐in.

The models are compared in terms of two widely used metrics in evaluating cause‐of‐death assignment: The accuracy of the predicted top cause of death and the CSMF accuracy. The CSMF accuracy metric is widely applied to evaluate how closely the estimated population distribution of causes of death matches the true distribution. It is defined as

(15) $$\begin{array}{ll} {\text{CSMF}}_{\text{acc}}(\hat{\pi }) & =1-\frac{\sum_{c=1}^{C}|{\hat{\pi }}_{c}-\pi_{c}|}{2\{1-\underset{c}{\min}\pi_{c}\}}, \end{array}$$

where $\pi_{c}$ denotes the true CSMF from the target dataset. This metric measures the discrepancy between the estimated and actual CSMF, and is scaled to be between 0 and 1, where higher values indicate better performance in estimating prevalence. We note that these two metrics do not directly measure the goodness of fit of the tensor decomposition. However, we focus on these two metrics since they are of more direct interest when utilizing different tensor decomposition models to perform cause‐of‐death assignment.

**FIGURE 2:**
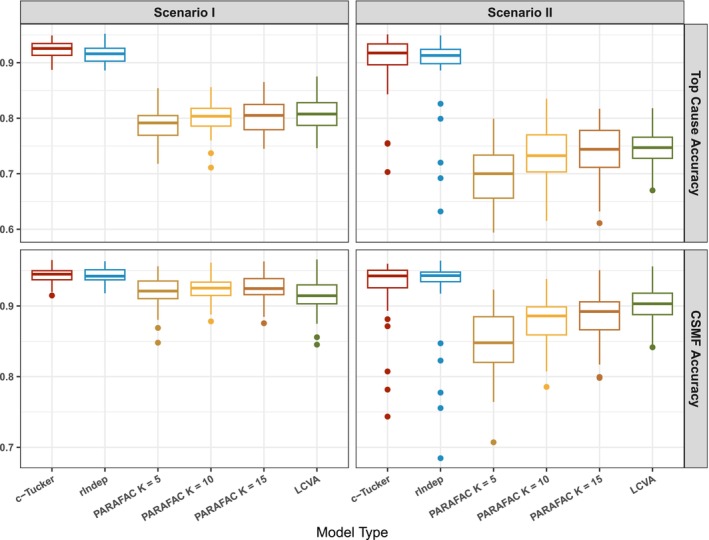
Top cause accuracy (top row) and CSMF accuracy (bottom row) for the two simulation scenarios, comparing the c‐Tucker model, the **r**‐group independent PARAFACs model, the PARAFAC with K=5,10,15 and LCVA with K=10 on the 50 simulated datasets.

Figure 2 summarizes the accuracy measures for different models. Performance of standard PARAFAC generally improves as K gets larger. Both the c‐Tucker and r‐group independent PARAFACs demonstrate higher accuracy compared to all standard PARAFAC models in both scenarios. Furthermore, the c‐Tucker and r‐group independent PARAFACs are more robust to distribution shift across datasets than the PARAFAC and single‐domain LCVA, with improvements evident in both the mean and variance of accuracy. While distribution shift is not intentionally accounted for in our model, this scenario shows the pitfalls of more rigid models (e.g., PARAFAC) when they fail to approximate the complex data distribution.

## 6 Analysis of PHMRC Gold‐Standard Dataset

In this section, we comprehensively evaluate our proposed models using the PHMRC gold‐standard VA dataset [21]. The dataset includes 7841 adult deaths across 34 distinct causes. This dataset has been processed into 168 binary symptoms following McCormick et al. [5]. We perform random sampling with replacement from the PHMRC dataset, selecting 80% of the data as training samples. To ensure that this subset is representative of the entire population, we set the prevalence, $\pi^{\left(1\right)}$, to match the true prevalence observed across the full dataset. We then generate target datasets by first sampling the target prevalence $\pi^{\left(0\right)}$ from Dir(1,…,1) and resampling the remaining observations not in the training data with replacement to match the target prevalence. We repeat the process to form 50 target datasets and model fittings are carried out in the same way as described in the previous section.

In the rest of this section, we first describe how we choose the number of latent components for the c‐Tucker and r‐group independent PARAFACs models in Section 6.1. Then we take the c‐Tucker model as a case study and further delve into the estimated latent parameters and the interpretation of model components. We discuss the symptom grouping structure learned from data in Sections 6.2 and 6.3. Finally, we assess the cause‐of‐death assignment accuracies of the proposed model and compare them with existing VA methods in Section 6.4.

### 6.1 Selecting the Number of Latent Components

In order to select a reasonable K and r, we first fit both models solely on the training data using large values for K and r, setting K=r=10. We select the smallest r that captures at least 95% of the group variation. For the c‐Tucker model, we choose r=8, as the first eight groups were utilized in more than 5% of posterior samples. Similarly, we set r=6 for the r‐group independent PARAFACs model, as the first six groups meet this threshold. The number of latent classes K is selected similarly. A small K is usually preferable in practice to avoid overfitting and reduce computational complexity. We choose K so that the majority of latent classes with a high utilization rate can be captured when fitting the models on labeled data. We let K=4 for the c‐Tucker model and K=5 for the r‐group independent PARAFACs model. This choice is sufficient to capture over 80% of latent classes utilized in at least 5% of posterior samples. More than 99% of such latent classes can be captured by letting K=6 for both models, but in practice, we find larger K usually leads to overfitted models and worse classification performance. As for the c‐Tucker model, we set h=3 for the high‐level decomposition of the mixing weights since we allow only eight symptom groups. Graphical summaries of the latent class utilization are included in the Supporting Information. When fitting the standard PARAFAC model, we select K=10 based on the recommendation of [10] on the same dataset.

We note that selecting the number of components in a mixture model is notoriously difficult [22, 23, 24, 25], and more importantly for our task, the optimal estimation of p(X|Y) does not always translate to the optimal classification performance, as the latter depends more directly on approximating the relative magnitude of p(Y|X) across different causes. Having more latent components can increase the risk of overfitting for some causes and decrease the classification performance. In our experiments, model selection criteria, such as WAIC and leave‐one‐out cross validation, tend to select more components and lead to high computational cost and inferior performance in cause‐of‐death assignment. Cross validation may also be used to find the optimal number of latent classes for a given classification metric, but the computational cost for cross validation on a grid of latent dimension choices is high and impractical for routine use. Thus, while the procedure we take here is ad hoc, we believe it is a practical approach that yields a small number of latent components to use and can be adopted by practitioners.

### 6.2 Grouping of Symptoms

**FIGURE 3:**
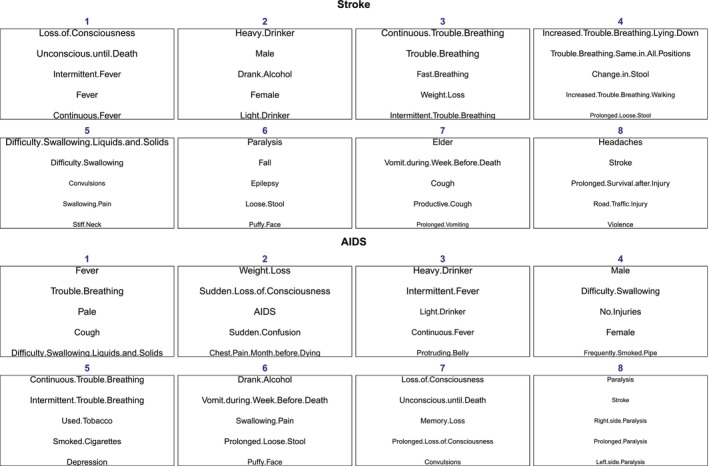
The group structure of key symptoms from the c‐Tucker model for two major causes of death: Stroke and AIDS. Within each group, symptoms are ordered by their probability of being in that group, listed from highest to lowest. Due to space, only the first five symptoms are shown. For symptoms with the same probability of being in the group, we sort them based on the empirical probability of observing the symptom given the cause, that is, $p(X_{ij}=1|Y=c)$. The size of the label is also proportional to this empirical conditional probability. The eight groups are re‐ordered to have decreasing average empirical conditional probabilities of individual symptoms.

We now illustrate the estimated symptom structures based on the c‐Tucker model in one resampled dataset based on the PHMRC data. Figure 3 presents a summary of key symptom groups for deaths due to two major causes in the PHMRC dataset: Stroke and AIDS. We determine the symptom group membership based on the posterior mode of the group indicator matrix s. Symptoms within each group are ordered by the posterior probability of each symptom being within that group, that is, $p(s_{cj}={ŝ}_{cj})$, in decreasing order. We focus on symptoms with high group assignment probabilities because when this probability is close to one, the symptom serves as an anchor to the latent symptom cluster, which has been shown to be key to ensure the identifiability of latent representations [26, 27]. Thus, Figure 3 can be viewed as a representation of key symptom “topics” within each of the eight groups, analogous to the topic modeling literature. In general, our model does not enforce the existence of anchor symptoms for all groups by design, but we have found such anchor symptoms with high group assignment probabilities in our numerical analysis of the PHMRC data consistently. We also note that being in the same group does not determine the sign of pairwise correlations. Rather, symptoms in the same group tend to vary together following the K group‐level sub‐profiles.

**FIGURE 4:**
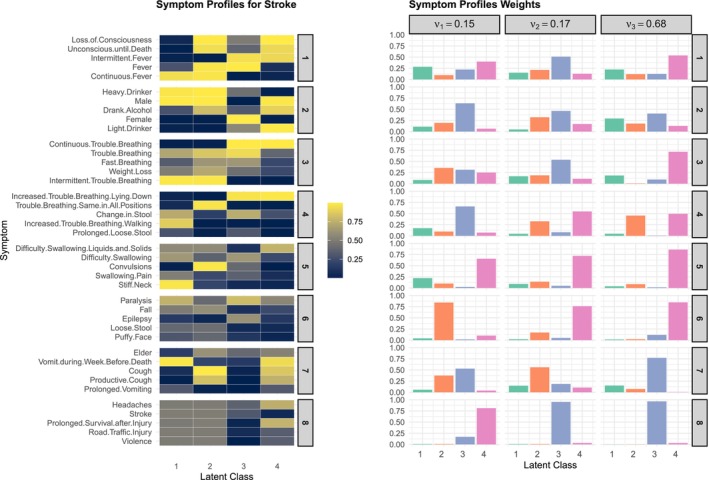
Posterior mean of latent parameters for stroke estimated from the c‐Tucker model in one synthetic dataset. The left heatmap is the selected symptoms profile ϕ by latent class from eight symptom groups, with the group and symptoms matching the same order as Figure 3. The right barplots are the symptoms profile latent weights decomposed with ψ and corresponding ν.

We now take a closer look at latent symptom profiles and corresponding mixing weights through the posterior distributions of ϕ, μ, and ψ. Figure 4 illustrates these relationships for the cause of death attributed to stroke estimated by the c‐Tucker model. On the left, symptom sub‐profiles $\phi_{c}$ are shown for the eight estimated symptom groups and the first five anchor symptoms within each group, as in Figure 3. The complete set of symptom profiles can be constructed by mixing the group‐level symptom sub‐profiles. In this example, $4^{8}=65,536$ expanded symptom profiles can be constructed, though many of which have a very low probability of occurrence.

The relative prevalence of the expanded symptom profiles is determined by the estimated weights in the right panel of Figure 3. Let c denote the index for stroke, the weights $\nu_{c}$ specifies h=3 set of random weights. In this example, $\nu_{c3}=0.68$ is the largest latent class on the group weights, indicating that around 68% of deaths due to stroke is expected to follow the third set of group‐level latent weights $\psi_{c3s}$ for each group s=1,…,8. While interpreting Figure 4 is selective by nature as only a subset of symptoms is shown due to space limitation, we can still observe interesting patterns that match clinical characterizations of stroke deaths. For this largest cluster, the first symptom group is dominated by latent Class 4, the second symptom group is mostly split between the latent Classes 1 and 3, and so forth. Focusing on the symptom profiles with high conditional probabilities, we can characterize this cluster of stroke deaths as having high probability reporting symptoms including loss of consciousness, unconsciousness until death, continuous trouble breathing, and having difficulty swallowing, which are all common comorbidities of severe stroke. They are also expected to split about evenly between having trouble breathing lying down and in all positions, likely depending on the severity of the stroke. The risk of experiencing paralysis is also elevated slightly based on the sixth group of symptoms and the deaths are slightly more likely to be females than males based on the second group of symptoms. In comparison, for example, in the next largest cluster with $\nu_{c2}=0.17$, the deaths are more likely to report symptoms including fever, cough, productive cough, and so forth, which may be related to medical complications such as pneumonia, which are also common among stroke deaths.

### 6.3 Grouping of Causes of Death

Estimating cause‐specific symptom grouping also allows us to compare causes of death in terms of how symptoms cluster. While we do not assume any information sharing across causes by design, we observe similarities across causes in terms of how symptoms are grouped. For example, comparing stroke and AIDS in Figure 3, some symptom groups are similar, for example, symptom clusters related to breathing problems and loss of consciousness, while some other groups are distinct for each specific cause.

**FIGURE 5:**
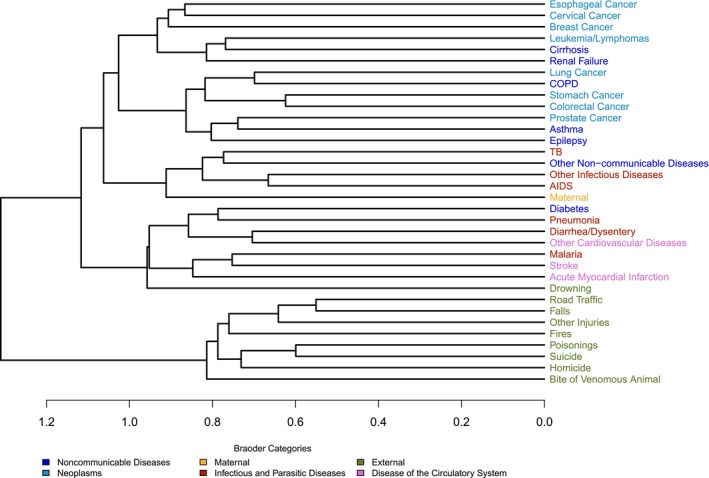
The cause of death dendrogram from the c‐Tucker model. The *x*‐axis represents dissimilarity between cause of death labels, calculated as 1—adjusted rand index (ARI), where ARI measures clustering similarity of symptoms across causes based on the posterior mode of group assignments in $s_{cj}$. The cause of death labels are colored by the six broader categories shown in the legend.

We further explore the similarities of symptom groups across different causes by applying hierarchical clustering to the causes using the estimated s matrix. Figure 5 shows the dendrogram of all causes of death based on the similarity of s. The clustering structure reflects dissimilarity between causes, calculated as 1—adjusted rand index (ARI), where ARI quantifies the similarity in symptom clusters across different causes of death [28]. The clustering corresponds very closely to the medically meaningful broader categories of causes. This suggests that the partitions of symptom groups estimated by the c‐Tucker model are generally similar among related causes.

### 6.4 Model Performance Comparison

To assess the model performance, we compare the results with single‐domain LCVA as in the simulation study and InSilicoVA algorithm [5]. InSilicoVA is a widely used VA method by practitioners. While it has been shown in Li et al. [10] that LCVA outperforms InSilicoVA in a variety of contexts, we include InSilicoVA as a practical baseline. Other recent VA algorithms have been shown to perform worse than or comparably to LCVA on the PHMRC dataset and thus we do not include them here for simplicity.

**FIGURE 6:**
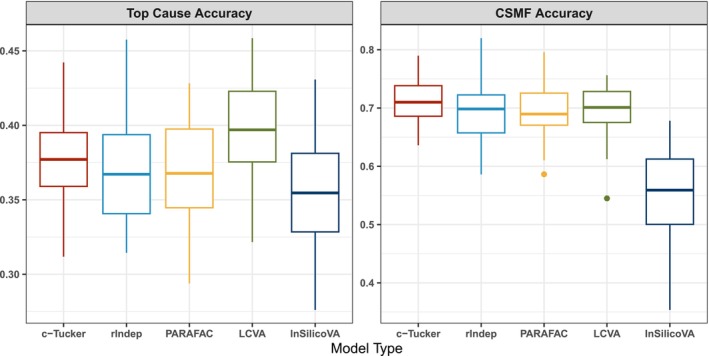
Boxplot of top cause accuracy (left) and CSMF accuracy (right) for different models on the 50 resampled target datasets.

Figure 6 shows the top cause accuracy and CSMF accuracy across different models on 50 resampled target datasets. For both metrics, the simplest model, InSilicoVA, exhibits the lowest overall accuracy and the highest variance. The c‐Tucker model shows slightly lower top cause accuracy than LCVA but outperforms all methods in CSMF accuracy. We caution against overinterpreting the relative performance of methods on the PHMRC dataset, as it is a single dataset with limited sample size and known issues [29]. Nevertheless, the numerical analysis shows that both the c‐Tucker and r‐group independent PARAFACs decomposition achieve comparable performance to existing methods and have the potential to be used as a routine analysis method for VAs. Overall, we prefer c‐Tucker model over the other tensor decomposition approaches since it provides more structured interpretation of the relationship across groups. However, as discussed before, the task of estimating symptom distribution and the task of classification are related but different. The r‐group independent PARAFACs model performs similarly to the c‐Tucker model in the numerical analysis we conducted, indicating that it can provide a reasonable approximation of the joint symptom distribution for the purpose of cause‐of‐death assignment and is also a reasonable approach in practice.

## 7 Discussion

In this paper, we examined the task of modeling VA data through the lens of conditional tensor decomposition. We proposed two dimension‐grouped tensor decomposition models for VA data, the c‐Tucker decomposition and group independent PARAFACs. Both models can identify multiple latent symptom groups and their corresponding profiles, offering a richer and more robust alternative to models based on standard latent class model formulation. Our approach not only captures complex and meaningful dependence structures among symptoms, but also enhances interpretability of the latent parameters by utilizing a more parsimonious parameterization to represent the symptom distributions. Our models also lead to robust improvements in cause‐of‐death assignment performance at both individual and population levels.

There are several limitations of the proposed model. First of all, while the symptom group structures show strong similarities across related causes of death, we did not explicitly encourage such structures in our model. We do not expect such information sharing to significantly improve predictive performance in the current study when the cause‐specific sample sizes are moderate to large, but we think it is a promising direction for future research and can be very useful to consider with rare diseases and unbalanced datasets. Second, in this paper, we only focus on the case of label shift between training and target datasets. The proposed methods can be naturally extended to deal with distribution shift in p(X|Y) in a multi‐source domain adaptation setting discussed in Li et al. [10]. Third, while our model provides a more flexible characterization of the symptom dependence, it comes at the cost of a heavier computational burden. While computation is feasible on a single laptop for the analysis considered in this paper, it can be challenging when scaling up to large datasets from national mortality surveillance systems, or when cross validation is desired to fine‐tune hyperparameters in the model. More work needs to be done to enable distributed modeling and inference for big data. Fourth, the dimension‐grouped decompositions we consider in this paper can also be extended to incorporate mixed membership models, where each symptom may contribute to multiple groups. Such models have been proposed recently in the literature [30, 31], which is a promising direction of future work. The structures of the latent symptom groups and profiles could also be extended in various ways. For example, when domain knowledge exists about broad symptom grouping, we may restrict the symptom grouping to be within the broad groups only to further simplify the process of choosing the latent dimensions. Lastly, the latent group structures we consider in this paper may be further condensed into lower‐dimensional summaries by adding more model components such as sparsity‐inducing priors. It is an important future direction of research to gain more insight into the interpretability and expandability of VA algorithms, especially as more and more black‐box classification tools are available in the era of artificial intelligence. We leave these challenges to future work.

## Funding

YZ and ZRL were supported by Grant R03HD110962 from the Eunice Kennedy Shriver National Institute of Child Health and Human Development (NICHD), and in part by the Bill & Melinda Gates Foundation. The findings and conclusions contained within are those of the authors and do not necessarily reflect positions or policies of the Bill & Melinda Gates Foundation.

## Conflicts of Interest

The authors declare no conflicts of interest.

## Supporting information




**Data S1.** sim70475‐sup‐0001‐Supinfo.

## Data Availability

The data that support the findings of this study are openly available in Population Health Metrics Research Consortium Gold Standard at https://ghdx.healthdata.org/record/ihme‐data/population‐health‐metrics‐research‐consortium‐gold‐standard‐verbal‐autopsy‐data‐2005‐2011.
