# Supplementary material for: Bayesian Tensor Decomposition for Clustering Latent Symptom Profiles for Verbal Autopsy Data
Source: Stat Med. 2026 Mar 3;45(6-7):e70475. doi: 10.1002/sim.70475 (PMC12956427; doi:10.1002/sim.70475)
Supplement: Supplementary file 1 — Data S1. sim70475‐sup‐0001‐Supinfo. [file SIM-45-0-s001.pdf]

Supplementary Materials for "Flexible Bayesian Tensor Decomposition for Verbal Autopsy Data"

Contents

1 Relationship to Tucker decomposition 1

2 Additional simulation results with misspecified latent dimensions 3

3 Additional analysis of PHMRC Gold-Standard Dataset 4

3.1 Selecting the number of latent components 4

3.2 Pairwise symptom dependence 5

3.3 MCMC convergence 6

1 Relationship to Tucker decomposition

The Tucker decomposition expresses a tensor as the product of a core tensor and factor matrices along each mode. The core tensor captures the interactions between the modes, while the factor matrices represent the mode-specific transformations. Specifically, we can express the probability tensor $p(X_{i1} = x_1, ..., X_{ip} = x_p \mid Y_i = c)$ in the form of K-component Tucker decomposition as below:

$$p(X_{i1} = x_1, ..., X_{ip} = x_p \mid Y_i = c) = \sum_{k_1=1}^{K} ... \sum_{k_p=1}^{K} \lambda_{ck_1,...,ck_p} \prod_{j=1}^{p} \phi_{ck_j,\, j}^{x_j} (1 - \phi_{ck_j,\, j})^{1-x_j}. \quad (1)$$

Here $\boldsymbol{\lambda}_c$ is a K^p-dimensional core probability tensor. The Tucker decomposition provides a more detailed and comprehensive representation of the tensor by capturing mode-specific transformations and inter-mode interactions. However, the interpretability can be more challenging due to the higher dimensionality of the factor matrices and the core tensor. The computational complexity is also significantly increased. Tucker decomposition has not been explored in the context of VA and it is likely not an effective model given the high dimensionality of VA data.

A graphical representation for the structure of latent variable Z and indicators X, conditional on a single cause of death, under different models is summarized in Figure 1.

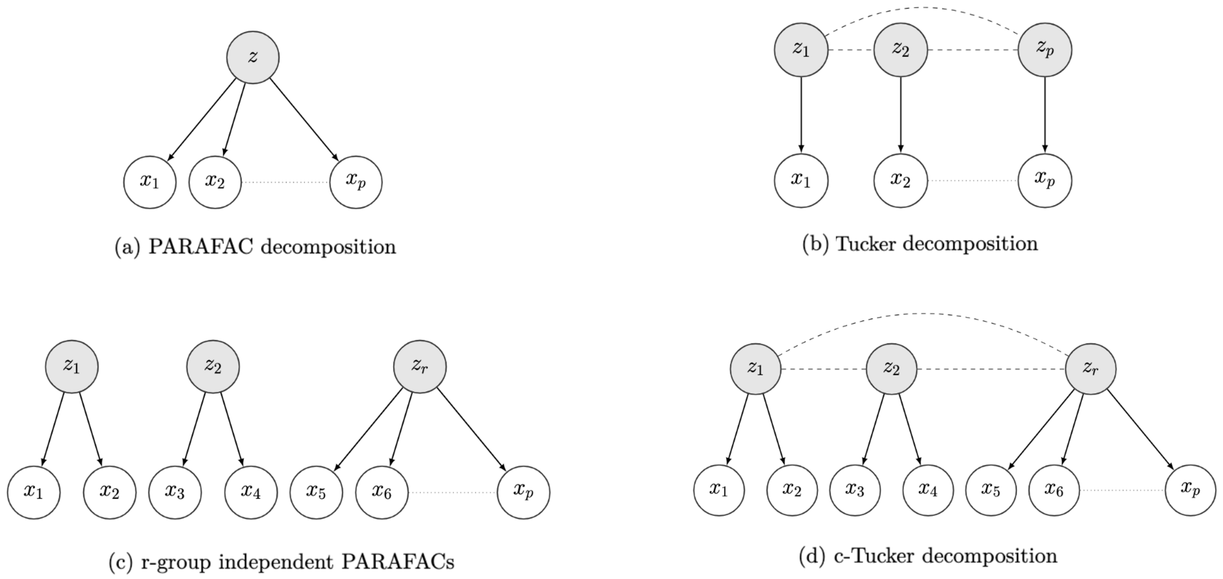

(a) PARAFAC decomposition

(b) Tucker decomposition

(c) r-group independent PARAFACs

(d) c-Tucker decomposition

Figure 1: Dependence structures of latent variable Z and indicators X under PARAFAC decomposition, Tucker decomposition, r-group independent PARAFACs and c-Tucker decomposition.

2 Additional simulation results with misspecified latent dimensions

In addition to the simulation analysis described in the main paper, we consider another scenario with misspecified latent dimensions. We generate data according to the proposed c-Tucker model with $r = h = k = 5$, and then fit the proposed models with the latent dimensions to be the same as in the main paper, i.e., $K = 3$, $r = 5$ and $h = 3$. Figure 2 shows the model results. The accuracy measures are uniformly lower than the correctly specified case of Scenario I in the main paper, but the relative performances remain the same.

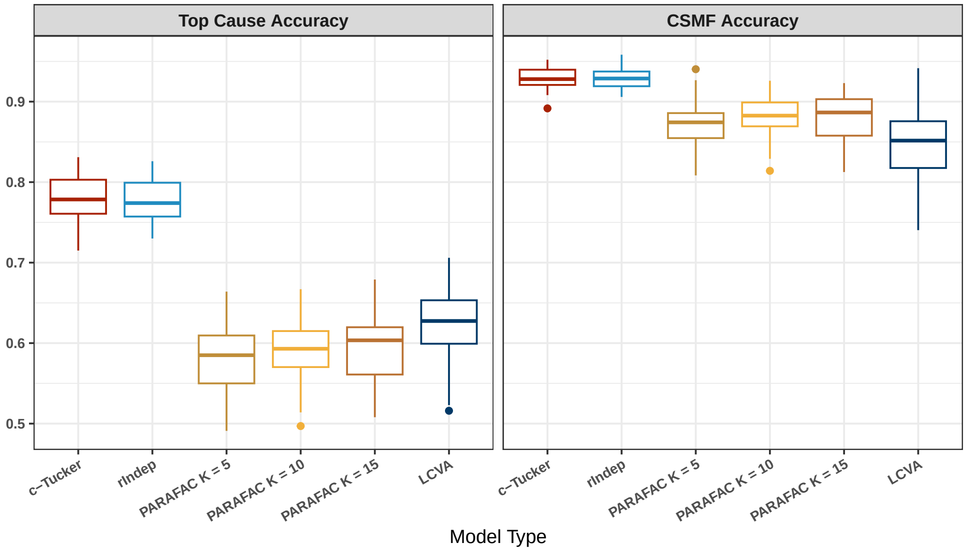

Figure 2: Top cause accuracy and CSMF accuracy for the simulation with data generated under $r = h = k = 5$, comparing the c-Tucker model, the r-group independent PARAFACs model, the PARAFAC with $K = 5, 10, 15$ and LCVA with $K = 10$ on the 50 simulated datasets.

3 Additional analysis of PHMRC Gold-Standard Dataset

3.1 Selecting the number of latent components

In order to select a reasonable K and r, we first fit both models solely on the training data using large values for K and r, setting $K = r = 10$. We select the smallest r that captures at least 95% of the group variation. Figure 3 illustrates the fraction of times each group is utilized from the posterior samples of $\boldsymbol{s}$ for each cause of death, along with average fractions over all causes. For the c-Tucker model, we choose $r = 8$, as the first 8 groups were utilized in more than 5% of posterior samples. Similarly, we set $r = 6$ for the r-group independent PARAFACs model, as the first 6 groups meet this threshold.

The number of latent classes K is selected similarly. A small K is usually preferrable in practice to avoid overfitting and reduce computational complexity. We choose K so that the majority of latent classes with a high utilization rate can be captured when fitting the models on labeled data. Figure 4 shows the proportions of posterior samples where each latent class is utilized, for each symptom group and each cause of death. We let $K = 4$ for the c-Tucker model and $K = 5$ for the r-group independent PARAFACs model. This choice is sufficient to capture over 80% of latent classes utilized in at least 5% of posterior samples. More than 99% of such latent classes can be captured by letting $K = 6$ for both models, but in practice, we find larger K usually leads to overfitted models and worse classification performance. As for the c-Tucker model, we set $h = 3$ for the high-level decomposition of the mixing weights since we allow only 8 symptom groups.

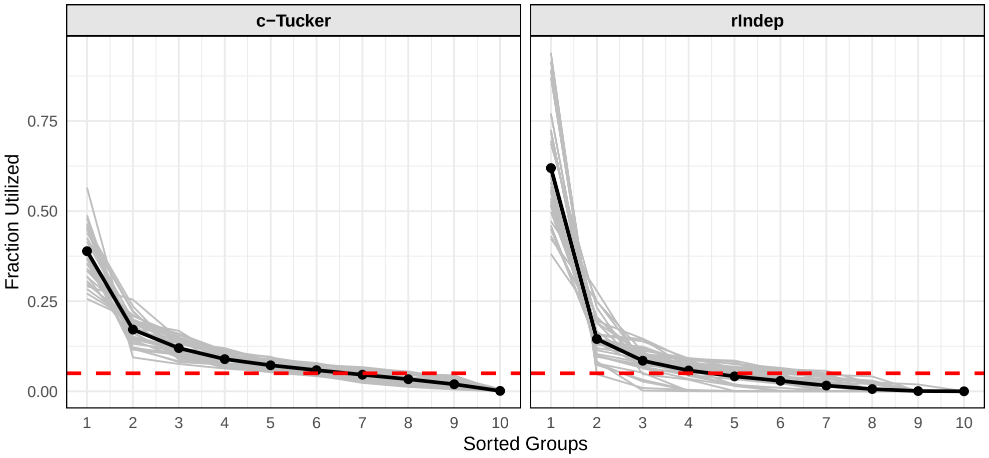

Figure 3: The fraction of times each group is utilized in the posterior samples by the c-Tucker and r-group independent PARAFACs models when there are $K = 10$ latent classes. The groups are sorted by the frequency when they are occupied. Each grey line represents one cause of death. The black line represents the simple average across all the causes.

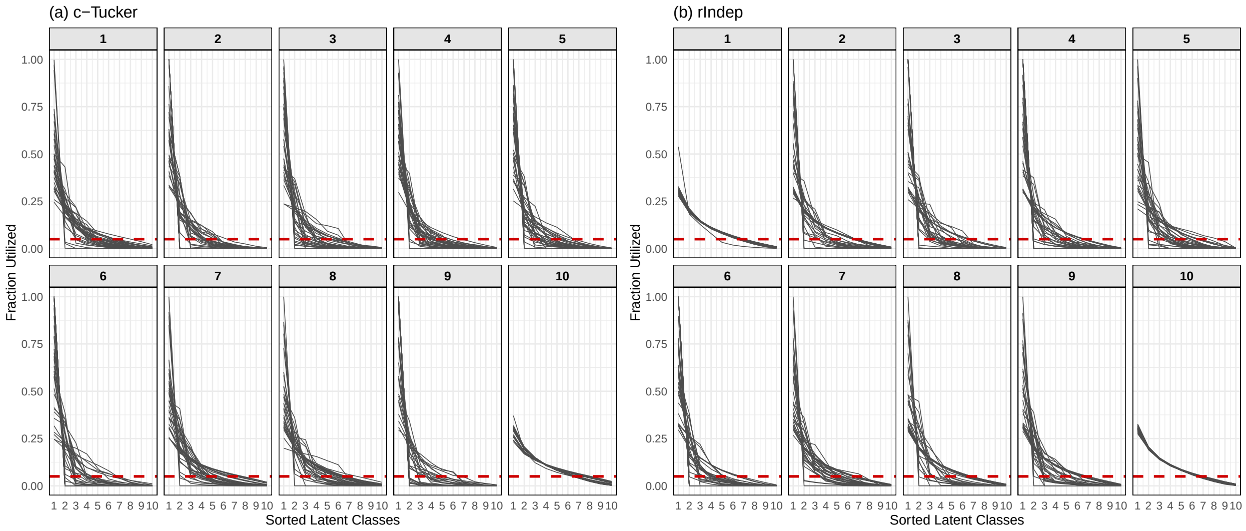

Figure 4: The fraction of times each latent class is utilized in the posterior samples by the c-Tucker and r-group independent PARAFACs models when there are $r = 10$ groups. The latent classes are sorted by the frequency when they are occupied in each group. Each green line represents one cause of death.

3.2 Pairwise symptom dependence

In addition to the cause-of-death assignment performance, we also compare different models in terms of the implied pairwise dependence of symptoms. Figure 5 compares the empirical Kendall's τ of symptom pairs for stroke from one synthetic dataset and the posterior means from c-Tucker, r-group independent PARAFACs, and the standard PARAFAC. Symptoms with missing proportions larger than 0.1 or those without variation are excluded. The estimated Kendall's τ from all three models track closely with the empirical values. The c-Tucker model captures more subtle grouping structures compared to the other two models.

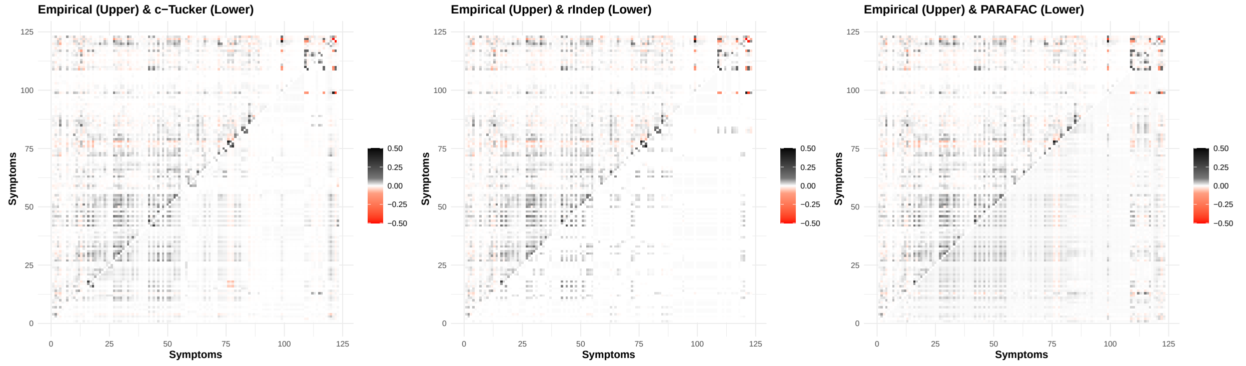

Figure 5: Empirical Kendall's τ of symptom pairs for stroke from one synthetic dataset (upper triangle) and posterior means (lower triangle) from the c-Tucker model (left), the r-group independent PARAFACs model (middle) and the PARAFAC model (right).

3.3 MCMC convergence

Figures 6, 7 and 8 show the trace plots of the estimated CSMF $\boldsymbol{\pi}^{(0)}$ in the target dataset from three models of one synthetic PHMRC dataset.

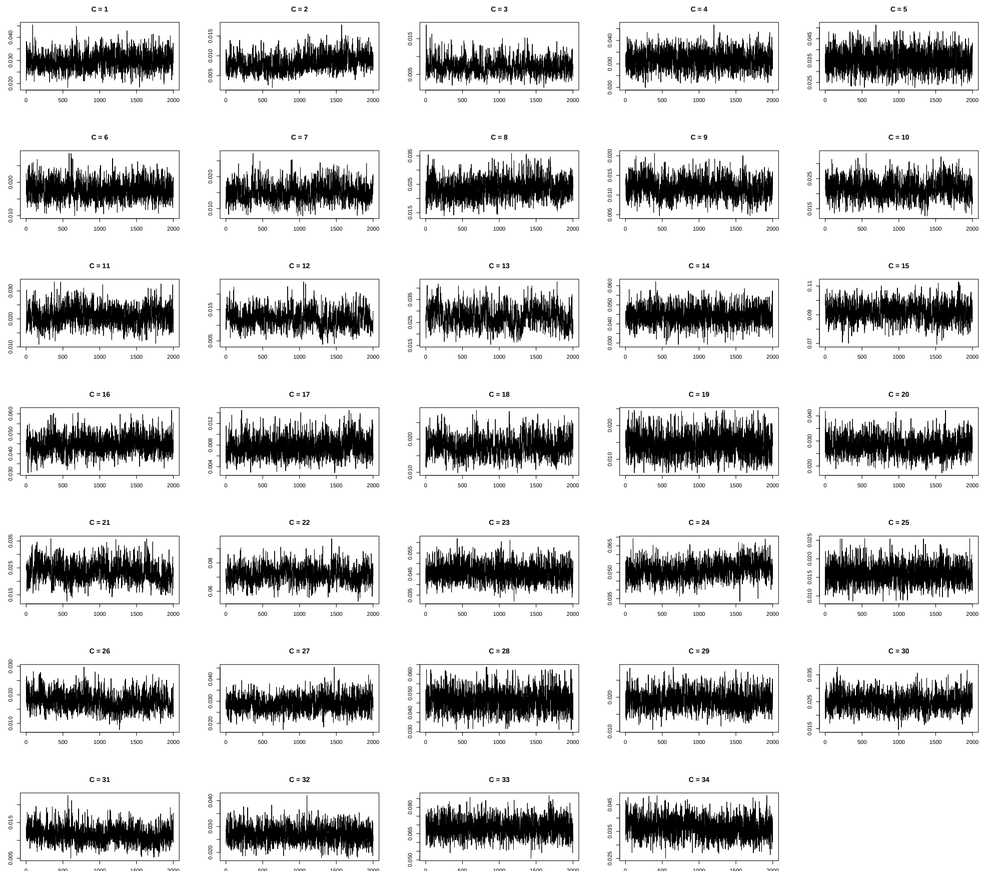

Figure 6: Traceplots of the CSMF in the target dataset, $\boldsymbol{\pi}^{(0)}$ from the c-Tucker model in one synthetic PHMRC dataset.

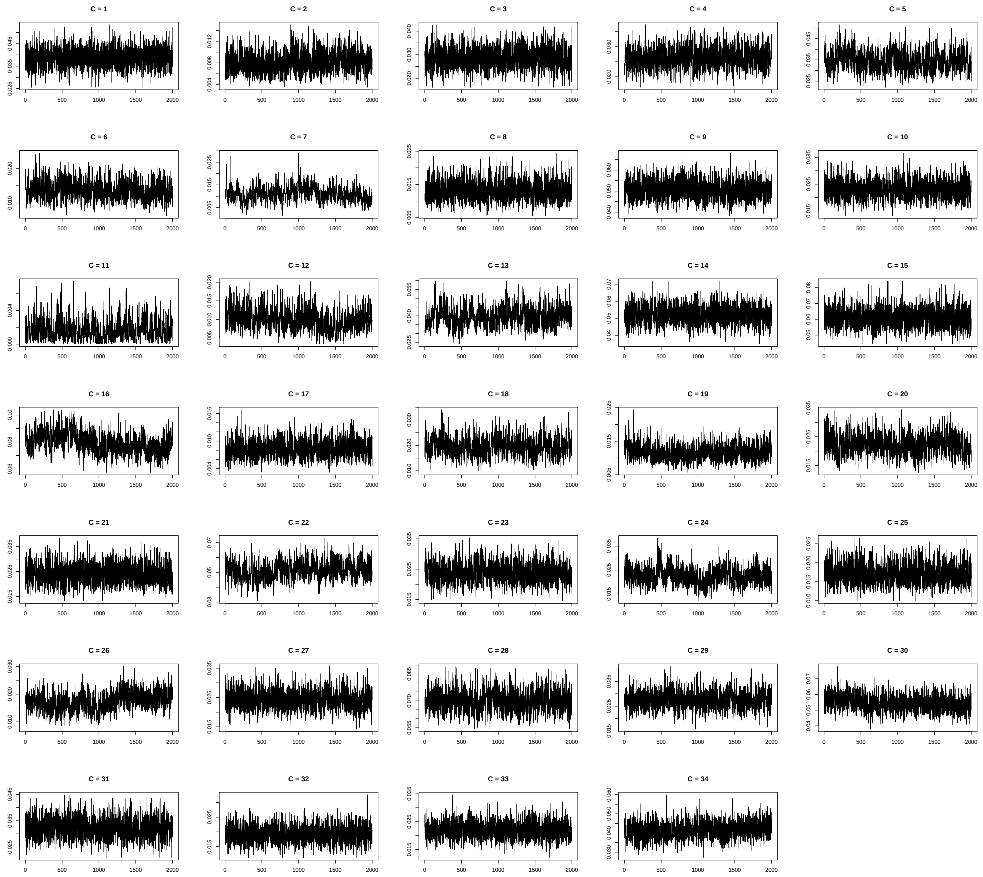

Figure 7: Traceplots of the CSMF in the target dataset, $\boldsymbol{\pi}^{(0)}$ from the r-group independent PARAFACs model in one synthetic PHMRC dataset.

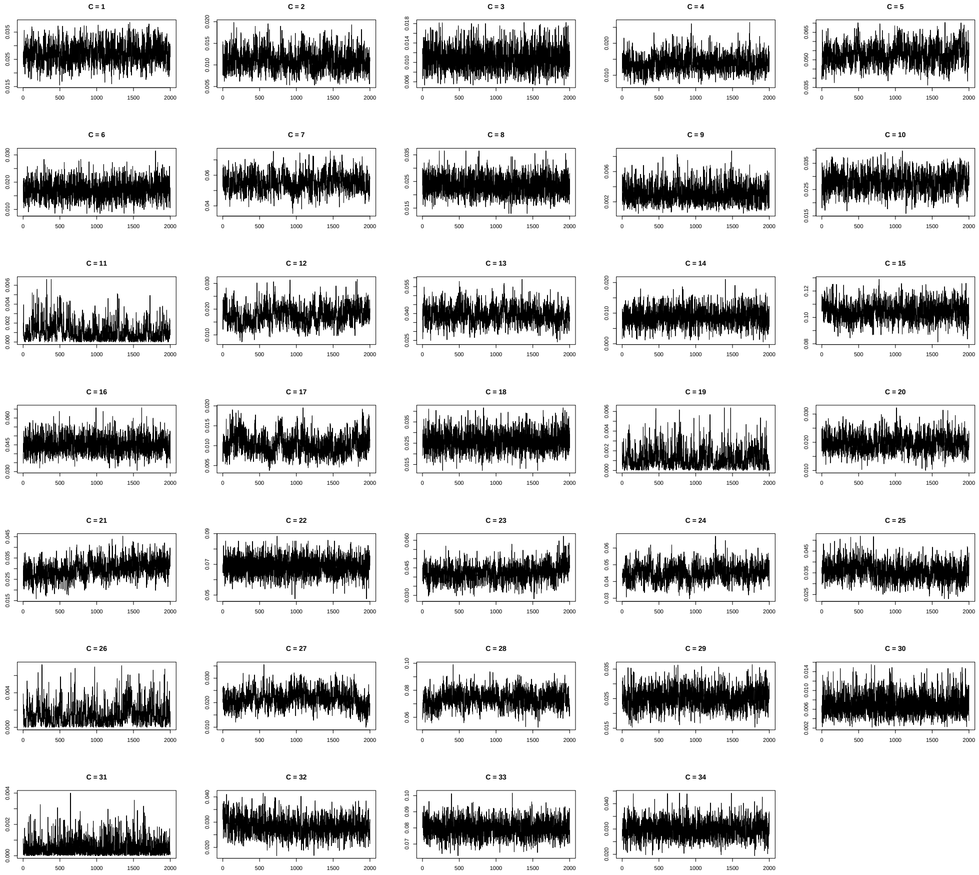

Figure 8: Traceplots of the CSMF in the target dataset, $\boldsymbol{\pi}^{(0)}$ from the PARAFAC model in one synthetic PHMRC dataset.
